# Case in Which the Duodenum Was Imperforate, and Other Visceral Peculiarities Existed in a Child

**Published:** 1823-06

**Authors:** H. Jeffreys


Art. III.—*Case in which the Duodenum was imperforate, and other Visceral Peculiarities existed in a Child.* By H. Jeffreys, Esq.

I was desired to examine the body of a male infant, who had died on the ninth day from its birth. I was informed that the mother had gone her full time. The child, when born, was in a state of considerable emaciation; and the anus was observed to be unusually small. It had vomited incessantly till its death, and, besides the breast-milk and other food, a large quantity of green slimy matter had been continually rejected from the stomach. Soon after birth there had been a discharge of meconium from the anus, and afterwards some small, green, slimy evacuations took place; but the child had no stool having any resemblance to fæces, and a clyster that had been administered was returned unchanged. Its voice was particularly puny and weak; the face and eyes were frequently convulsed and distorted; and it continued to emaciate till its decease.

I opened the body the following day. There was no omentum. The stomach was very much distended; and, when cut into, a large quantity of green fluid escaped. Its villous coat was covered with large red patches, apparently arising from inflammatory action. The intestines, in colour, size, and appearance, resembled a cluster of earth-worms. The root of the mesentery, instead of adhering to the vertebræ, was attached to the cardiac extremity of the stomach, from whence the intestines hung by it, as by a stalk or pedicle. The head of the colon had no attachment to the ileum, but was fixed to the root of the mesentery and stomach, from whence the bowel took its regular course to the rectum. The duodenum was twisted round the root of the mesentery and the head of the colon; and, on passing a probe from the stomach into that gut, it was found to be completely impervious about half an inch from its origin.

*Clarges-street; April,* 1823.

